# Genes and their single nucleotide polymorphism involved in innate immune response in central nervous system in bacterial meningitis: review of literature data

**DOI:** 10.1007/s00011-018-1158-3

**Published:** 2018-05-12

**Authors:** Ewelina Gowin, Danuta Januszkiewicz-Lewandowska

**Affiliations:** 1 Family Medicine Department, Poznan University of Medical Sciences, Przybyszewskiego 39, 60-355 Poznan, Poland; 2 Department of Oncology, Hematology and Bone Marrow Transplantation, Poznan University of Medical Sciences, Poznan, Poland; 3 Department of Medical Diagnostics, Poznan University of Medical Sciences, Poznan, Poland

**Keywords:** Innate immune response, Bacterial meningitis, Central nervous system

## Abstract

**Background:**

There are many studies analysing the effect of SNPs in genes coding proteins which are involved in innate immune response on susceptibility to invasive bacterial disease. Many of them gave inconclusive results. Regarding the complexity of immune response and cooperation between particular elements, number of SNPs may have a cumulative effect on the susceptibility to bacterial meningitis.

**Findings:**

In most studies cooccurrence of several SNPs was not analysed. These studies were performed on small groups of patients and usually only few SNPs were checked simultaneously. Additionally, comparison of the results across the studies is hard to conduct. We hypothesise that the number of variants of genes involved in innate immune response plays a role in susceptibility to bacterial meningitis. However, the role of toll-like receptors and other part of innate immune response in the eradication of bacteria, and initiation of the inflammatory response in CNS need further studies.

**Conclusion:**

Large multicentre studies assessing multiple SNPs in patients with microbiologically proven pneumococcal or meningococcal meningitis are needed to find real genetic risk factors for developing bacterial meningitis. This is necessary to design more effective treatment and prevention strategies for severe infections.

## Introduction

Bacterial meningitis (BM) is one of the most devastating infections in childhood with a high rate of mortality. Despite the common use of vaccination, the leading causes of bacterial meningitis are *Streptococcus pneumoniae* and *Neisseria meningitidis* [1–3]. Carriage of these bacteria is prevalent in general population. The question is why some people are only asymptomatic carriers while others develop the devastating disease. Most patients with BM were previously healthy, not suffering from chronic diseases, and not diagnosed with immunodeficiency. There are several steps to develop bacterial meningitis [4]. The first one is the colonisation of mucous membranes. Then, after invasion through mucous membranes bacteria reach the blood. Even with the protective blood–brain barrier, pathogens can reach the central nervous system (CNS). The final step is an inflammatory process in the cerebrospinal fluid.

Immune response in CNS is unique. In cerebrospinal fluid (CSF) antigen presenting cells (APC) are not present and the subarachnoid cavity has no lymphatic drainage [5]. The goal of innate immunity in CSF is the recognition and response to a pathogen. It is different from peripheral innate response which cooperates with adaptive immune response in spleen and lymph nodes [6, 7]. In most cases pathogens enter CNS via blood. They were previously presented to peripheral immune cells, which are responsible for generating the adaptive immune response.

Immune response in CNS must be well balanced, and absence of adaptive immunity in CNS allows to protect neuronal tissue from damage caused by inflammatory processes. The inflammatory response in CSF is stimulated by pathogen-associated molecular patterns (PAMPs) and damage-associated molecular patterns (DAMPs). Among pattern recognition receptors (PRR) localised in leptomeninges and choroid plexus, toll-like receptors (TLR) are of crucial importance in antibacterial response [8–10].

Toll-like receptor was originally identified as a receptor essential for dorsoventral polarity in the early development of Drosophila [11]. TLRs are transmembrane PRRs; they constitute a large superfamily with interleukin 1 receptor (IL-1R) family. They are either expressed at the cell surface for extracellular ligand recognition or localised in endosomal compartments for the recognition of pathogen-associated nucleic acids. Among the characterised TLRs, TLR1, 2, 4, 5 and 6 are represented on the cell surface and recognise bacterial and fungal products, whereas TLR 3,7, 8 and 9 reside in the intracellular endosomes and specialise in the detection of pathogens’ nucleic acids [9, 12, 13]. Microglial cells express all TLRs identified to date; astrocytes express TLR 2, 3, and 9, neurons express TLR 3, 7, 8, and 9, and oligodendrocytes express TLR 2 and 3 [8]. The cytoplasmatic part of TLR possesses the domain with a high degree of similarity with IL-1R [9, 12]. Their horseshoe-like shape forms hetero or homodimers (M-shaped structure) to bind PAMPs. Signal transduction via TLR engages proteins such as myeloid differentiation factor 88 (MyD88), kinase IRAK (IL-interleukin-1R-associated protein kinases), kinase TAK1 (TGF-beta-activated kinase), kinase-binding protein TAK1, and TNF-receptor-associated factor 6 [14]. Within 4 h after TLR activation APCs, macrophages and natural killers (NK) are activated and production of cytokines (IL-1, IL-6, IL-12), TNF-alpha (TNF-α), interferon alpha/beta (IFN-α/β) occurs [14]. This activation cascade leads to pathogen elimination and inhibition of the inflammatory process through IL10 [14, 15]. Cytokines decrease the integrity of blood–brain barrier and increase leukocyte migration [6].

First data about TLR in pneumococcal meningitis were presented in paper by Echchannaoui et al. [16]. The authors shown more invasive diseases in TRL2-deficient mice. Nowadays, it is proven that three from ten different TLRs (TLR2, TLR4, and TLR9) are involved in inflammatory response to bacteria such as *S. pneumoniae* or *N. meningitidis* [17, 18].

The human TLR2 is stimulated by bacterial cell-wall components [20]. In a mouse model deficiency of TLR2 increases the severity of the pneumococcal meningitis [19]. TLR4 is found to be essential for lipopolysaccharide (LPS) signalling, but alone is not sufficient for LPS recognition. LPS is recognised by heterodimer of TLR4 and MD-2. A mutant of N. meningitidis which is LPS-lacking is not able to cause TLR4 dependent response [17].TLR4 is also stimulated by pneumococcal pneumolysin. TLR9 is an intracellular PRR which recognises phagocytosed unmethylated Cytosine–phosphate–Guanine (CpG) motives in bacterial DNA [9]. This protein is present in phagocytosing microglia and antigen presenting astrocytes in CSN. TLR9 activation triggers the MyD88-dependent pro-inflammatory pathway [19].

A recognition of certain PAMPs involves also a TLR-independent path. Of the best-characterised intracellular PRRs are the nucleotide-binding domain (NOD) proteins that recognise bacterial peptidoglycan. NOD-like receptors (NLRs) constitute a family of cytoplasmic PRRs, which contains more than 22 members [20–22]. NOD1 and NOD2 recognise peptidoglycans: NOD1 detects meso-DAP Gram negative and NOD2 recognises muramyl dipeptide, present in both Gram-positive and Gram-negative bacteria. NOD2 can also recognise single-strand RNA.

NLRs are characterised by a tripartite-domain organisation with a conserved NOD domain and leucine-rich repeats [23]. Their primary role is to recognise PAMPs and DAMPs to induce inflammasome formation and production of IL-1 and IL-18 via Caspase-1 activation. NLR binds to RIPK2 (receptor-interacting serine-threonine protein kinase 2) leading to nuclear factor-κB (NF-κB) and MAP kinase activation [23]. NOD-receptors are also involved in MyD88-dependent signalling cascade downstream of TLR2 and TLR4. Pneumococci can induce NF-κB activation through NOD2- and RIP2-dependent signalling [22].

Activation of NF-κB through a TLR or NOD-like receptor binding is considered to be the central regulator of the innate immunity. This mechanism is controlled by inhibitors which are encoded by genes NFKBIA, NFKBIE, and NFKBIZ (Nuclear Factor of Kappa Light Chain Gene Enhancer in B cells inhibitor) [24]. The degradation of NF-κB inhibitors, including IκB-α, IκB-ε, and IκB-ζ (encoded by the genes NFKBIA, NFKBIE, and NFKBIZ), leads to NF-κB translocation to the nucleus and gene transcription resulting in cytokine production [24].

TIRAP (toll–interleukin 1 receptor—TIR domain-containing adaptor protein) is an adaptor protein which is essential for the inflammatory response. TIRAP is involved in TLR2 and TLR4 signalling pathways leading to the activation of NF-κB, MAPK1, MAPK3 and JNK, and resulting in cytokine secretion and the inflammatory response. TIRAP also positively regulates the production of TNF-alpha and IL-6 [25].

There are no doubts that innate immune response is crucial in susceptibility to bacterial meningitis. Four Mendelian primary immunodeficiencies associated with impaired signalling of the TLR pathway have been reported with mutations in MyD88, interleukin-1 receptor-associated kinase-4 (IRAK4), nuclear factor-kappa B essential modulator (NEMO) and IκBα (nuclear factor of kappa light polypeptide gene enhancer in B-cells inhibitor, alpha) [26]. These diseases are caused by monogenic mutations [26]. The first described deficiency in TLR signalling is IRAK4 deficiency. This deficiency causes no response to TLR ligands including LPS. Cells after stimulation do not produce TNF-alpha, IL-1 beta, IL-12 p40, or IL-6 which results in recurrent staphylococcal and streptococcal infections [26, 27].

Mutations in genes coding proteins involved in innate immunity, although rare in general population, cause severe immunodeficiency manifesting with severe diseases in early childhood. On the other hand, the effect of single nucleotide polymorphisms (SNPs) in above-mentioned genes is not that harmful to a particular patient, but because SNPs are common—their global effect may be even more important.

Having in mind the complexity of the immune response, we see the need for analysis of SNPs in genes involved in naive immune response on bacterial meningitis. In this manuscript, we summarise the results from different studies to find out an association between SNPs in genes coding PRPs and susceptibility to bacterial meningitis (Table 1).

**Table 1:** Summary of the analysed studies

SNP analysed	Results	Study group	Disease	Age of studied population	Country	Authors
***TLR4*** **rs4986791** ***TLR4*** **rs4986790**	**no association**	185 IMD 770 HC	IMD MM	Adults and children	Austria	Biebl et al. [34]
***TLR4*** **rs4986790**	**no association**	1047 IMD 879 BD	IMD	Adults and children	UK	Read et al. [33]
***TLR 9*** **rs352140** ***TLR 9*** **rs5743836**	***TLR9*** **rs5743836—ProMM p** < **0.01**	**390 MM**	MM	Children	**Netherlands**	Sanders et al. [37]
*TLR4* **rs4986790**	*TLR4* **rs4986790 - ProIMD** *p* = **0.007** **in children** younger than 12 months	197 IMD 214 HC	IMD	Adults and children	Germany	Faber et al. [30]
*TIRAP* rs8177374 *NFKBIA* rs3138053 *NFKBIA* rs2233406 *NFKBIE* rs529948 *NFKBIZ* rs6165997 *TONSL* rs760477 *PTPN22* rs2476601	*NFKBIE* rs529948 SuPM p < 0.0001 all SNPs —no association in MM	372 PM 907 PB 1273HC 406 MM 272 HC	PM MM	children	Denmark	Lunbdo et al. [39]
*NFKBIL2* rs760477 *NFKBIL2* rs2306384 *NFKBIL2* rs4082353 *NFKBIL2* rs4925858 *NFKBIL2* rs10448143 *NFKBIL2* rs2170096 *NFKBIL2* rs2272658 *NFKBIL2* rs135258200 *NFKBIL2* rs4380978	*NFKBIL2* rs760477 SuIPD p = 0.0006 *NFKBIL2* rs4925858 SuIPD p = 0.003 remaining SNPs —no association	UK 275 patients 733 HC Kenya 687 patients 173 PB 550 HC	IPD	Adults Children	UK Kenya	Chapman et al. [40]
*TLR2* rs5743708 *TLR2* rs5743708 *TLR4* rs4986790	*TLR4* rs4986790 SuIMD p = 0.0472 *TLR 2* rs5743708 SuIMD p = 0.0003 *TLR 2* rs5743708 SuIPD p < 0.0001	59 IMD 18 MM 114 IPM 12 PM 66 HC	MM PM	Children	Europe	Telleria-Orriols et al. [31]
*TIRAP* rs8177374	*TIRAP* rs8177374 ProIPD p = 0.003	191 IPD 741 HC	IPD	Adults children	UK	**Khor and al** [25]
***TLR2*** **rs5743708** *TLR4* **rs4986790** *TLR4* **rs4986791**	no association	**99 IPD** **178 HC**	IPD	–	Belgium	**Moens et al**. [28]
*TLR2* rs5743708 *TLR4* rs4986790 *TLR4* rs4986791 *CD14*-159CC *FcγRIIA-R*/R131	*TLR4* **rs4986790 ProPM** p < 0.005 ***CD14-*159CC SuIPD p** < **0.05** ***FcγRIIA-R***/**R131 SuIPD p** < **0.001** **remaining SNPs —no association**	85 IPD 409 BD	IPD	Children	Australia	**Yuan and al** [29]
***TLR2*** ***TLR4*** **total nucleotide variation**	*TLR2* rs57437708 more common in patients p = 0.05	197 IMD 238 HC	IMD	Children	**UK**	Smirnova et al. [32]
*TLR2* rs5743708 *TLR4* rs4986790 *NOD1* rs6958571 ***NOD2*** **rs57**43293 *NOD2* rs2066847 *NOD2* rs2066844 *CASP1* rs2282659	*TLR4* **rs4986790 SuMM** ***NOD2*** **rs2066847 SuMM** remaining SNPs —no association	391 MM 82 PM 1141 HC	MM PM	Children	**Netherlands**	Van Wel et al. [36]
*TLR4* rs4986790 *TLR4* rs4986791	no association	252 MM 251 HC	MM	Children	Gambia	Allen et al. [35]
*TLR2* rs3804099 *TLR3* rs3775291 *TLR3* rs3775290 *TLR9* rs352139 *TLR9* rs352140	no association	218 MB 330 HC	BM	Children	China	Zhang et al. [38]

Bold values indicate significant results

*BD* blood donors, *BM* bacterial meningitis, *HC* healthy controls, *IPD* invasive pneumococcal disease, *IMD* invasive meningococcal disease, *MM* meningococcal meningitis, *PM* pneumoccoccal meningitis, *PB* pneumoccoccal bacteraemia, *SuPM* susceptibility to pneumococcal meningitis, *SuIPD* susceptibility to invasive pneumococcal disease, *SuIMD* susceptibility to invasive meningococcal disease, *SuMM* susceptibility to meningococcal meningitis, *ProIMD* protection against invasive meningococcal disease, *ProPM* protection against pneumococcal meningitis

A review of the medical literature from the last 16 years (2001–2016) was performed in the primary database PubMed, using MeSH descriptors and combinations of the descriptors “SNPs”, “bacterial meningitis”, “pneumococcal meningitis”, “meningococcal meningitis”. Excluded from the review were studies which included newborns, particularly due to the different meningitis-causing agents in this age group.

## TLR 2 and TLR4

There are two SNPs in *TLR2* widely described in the literature: rs4696480 and rs5743708. *TLR2* rs57437708 located in the Toll/IL-1 receptor region of TLR2 has an essential role in the formation of dimers [28]. The presence of this SNP induces a defective TLR2 dimerisation, and thus reduced response against meningococcal porin B [28, 29].

In the case of human *TLR4*, two common polymorphisms are found, rs4986790 and rs4986791, which are related to the hyporesponsiveness to LPS. Polymorphic variants are present in approximately 10% of Caucasian individuals [30]. As expected, people with these mutations are more frequently infected with Gram-negative bacteria than patients without mutations, and their condition is more severe. Although mutations are localised outside of the ligand-binding domain, they cause local conformational changes around the LPS-binding site, what results in decreased functional *TLR4* expression. That change interferes with TLR4 interaction with MyD88. *TLR4* rs4986790 mutated variant due to hyporesponsiveness to LPS may cause susceptibility to invasive meningococcal and pneumococcal infections [31].

Yuan et al. found a lower incidence of the *TLR4* rs4986790 polymorphism in a group of 85 Australian patients with invasive pneumococcal disease [29]. *TLR2* rs5743708 polymorphism was present in 4% children with pneumococcal meningitis and 7% of the control group (*p* = 0.04). This was a relatively small group of patients with invasive pneumococcal disease—not only meningitis. On the other hand, Moens et al. did not find any association between *TLR2* rs4696480, *TLR2* rs5743708, and *TLR4* rs4986790 SNPs and susceptibility to invasive pneumococcal infection in 99 Belgian patients [28].

Statistically significant differences in *TLR2* and *TLR4* SNP frequency were found by Telleria-Orriols [31]. In the study group, there were 59 children with meningococcal infection (18 with meningococcal meningitis) and 114 with pneumococcal infection (only 12 with meningitis) of Caucasian origin. 59% of patients with meningitis carried at least one copy of a risk allele. In the control group, the risk allele was found in 16.6% children (*p* < 0.0001). This study showed two times higher frequency of *TLR4* polymorphic variant in patients than in controls (23.7% vs 9.1%; *p* = 0.0472) [31].

Smirnova et al. analysed total nucleotide variation at *TLR4* and *TLR2* loci. The study group included 197 British patients with invasive meningococcal disease and only half of these patients had meningitis. Rare *TLR4* coding variants were markedly over represented in patients compared to controls. Similar situation was observed with rare variants of *TLR2* rs57437708, but this finding was not of statistical value [32].

In a study by Read et al. on 1047 British patients with proven meningococcal disease and 879 healthy controls, polymorphic variants of *TLR4* were detected in 6.5% of patients and 5.9% of controls [33]. The difference was not of statistical significance. Biebl et al. checked SNPs in *TLR4* in a group of Austrian survivors from meningococcal disease [34]. There were 185 patients with proven meningococcal infection and 770 healthy controls. Only 53% of patients had meningitis. Age of patients was widely scattered, ranging from 1 month to 74 years. *TLR4* rs4986790 was not significantly associated with the susceptibility to invasive meningococcal disease [34].

Age of studied population appeared to be important factor. Faber et al. in a group of children younger than 12 months found 1.55 times increased risk of meningococcal meningitis for *TLR4* rs4986790 variant carriers compared to controls [30]. Allen et al. in the study on meningitis did not show any association between *TLR4* rs4986790 and *TLR4* rs4986791 SNPs and susceptibility to meningococcal meningitis during epidemics in Gambian children [35].

Mutant alleles of *NOD2* were associated with decreased activation of NF-κB [22]. In vitro studies showed that *NOD2* is upregulated after exposure to *N. meningitidis*. Van Well et al. in the group of 391 children with meningococcal and 82 pneumococcal meningitis and 1141 controls showed that variants of *TLR4* rs4986790 and *NOD2* rs2066847 are significantly associated with meningococcal meningitis [36]. Polymorphic variants of *NOD2* rs2066847 were more common in patients with meningococcal meningitis but not with pneumococcal meningitis. No differences were observed for *TLR2* rs5743708.

## TLR9

*TLR9* rs352140 polymorphic allele creates an increased affinity for NF-κB which in turn increases the transcriptional activity of the gene leading to enhanced production of cytokines, what results in immune-mediated cochlear damage [37]. With respect to SNPs of *TLR9* (rs352139 and rs352140), no differences in frequencies of genotypes or alleles were observed among patients with bacterial meningitis and controls in a study by Zhang on Chinese children [38]. They could not find any significant differences when combined analysis of the two SNPs of *TLR9* was performed. Sanders et al. in a study on 390 paediatric survivors of meningococcal meningitis demonstrated a protective effect of *TLR9* rs352140 variants [38].

## TIRAP

Homozygous variants of *TIRAP* rs8177374 (which encode a leucine substitution at Ser180 of *TIRAP*) are rare in developing countries. It supports speculations that this homologous *TIRAP* rs8177374 variant may increase susceptibility to specific infectious diseases to such an extent that it may have selected itself out of population [25]. A recently discovered SNP in *TIRAP* rs8177374 impairs TLR2-mediated NF-κB signalling in reconstitution experiments [25]. The variant was less able to bind TLR2 in comparison with the wild one. Khor et al. showed a protective effect of heterozygous variant of *TIRAP* rs8177374 against pneumococcal infection, malaria, and tuberculosis [25].

## NFKBIA, NFKBIE and NFKBIZ

In the study by Lundbo et al. on a large group of Danish children with pneumococcal or meningococcal meningitis, the authors have shown the association between NFKBIE SNP and susceptibility to pneumococcal meningitis [39]. They checked seven SNPs also in other inhibitory factors (NFKBIA, NFKBIL2, NFKBIZ, TIRAP), but no association was found.

In the study group of Chapman et al., there were 275 British patients with 733 controls, and 173 African patients with 550 controls [40]. It was found that common NFKBIL2 polymorphisms were associated with susceptibility to invasive pneumococcal disease in European and African populations. The *NFKBIE* SNP was associated with increased risk of pneumococcal meningitis, but not bacteraemia. The remaining SNPs were not associated with susceptibility to invasive disease. None of the SNPs were associated with risk of invasive meningococcal disease or mortality [39, 40].

Altogether, we were able to summarise the literature on SNPs that affects the susceptibility to pneumococcal and meningococcal meningitis. There are many studies analysing the effect of SNPs in genes coding proteins which are involved in innate immune response on susceptibility to invasive bacterial disease (including bacterial meningitis). Many of them gave inconclusive results. These studies were performed on small groups of patients and usually only few SNPs were checked simultaneously. Regarding the complexity of immune response and cooperation between particular elements, number of SNPs may have a cumulative effect on the susceptibility to bacterial meningitis. In most studies cooccurrence of several SNPs was not analysed.

Additionally, comparison of the results across the studies is hard to conduct. The studies were performed on different groups of patients: some were conducted on patients with meningitis, and some on patients with sepsis. In cohort studies the percentage of patients with meningitis of proved bacterial aetiology was estimated at 10%. The diagnosis of meningitis is clear, but in some studies patients with bacteraemia or sepsis were also included. The gold standard for establishing the aetiology is the microbiological culture. Molecular detection of pathogen genetic material is also accepted, but in some studies meningococcal aetiology was based on the clinical picture—sepsis with severe purpura. Control groups were also different. Utilising the blood donors as the controls gives an opportunity to collect big group, but this method poses many disadvantages. In such a control group, the information about diseases in childhood is lacking. Most studies collected data from meningitis survivors, thus patients with the severest disease are excluded. Many studies were based on a genetic material from banks, thus researchers did not have detailed clinical data on past medical history of those children.

A large number of studies have been published to date, but those studies involved relatively small groups of patients. Many of the associations have not been reproduced in larger studies, so future studies should involve large group of patients with well-defined conditions. Large multicentre studies assessing multiple SNPs in patients with microbiologically proven pneumococcal or meningococcal meningitis are needed to find real genetic risk factors for developing bacterial meningitis, what is necessary to design more effective treatment and prevention strategies for severe infections.

## Abbreviations

APC: Antigen presenting cells
CNS: Central nervous system
CSF: Cerebrospinal fluid
DAMPS: Damage-associated molecular patterns
IκBα: Nuclear factor of kappa light polypeptide gene enhancer in B-cells inhibitor, alpha
IL: Interleukin
IFN-α/β: Interferon alpha/beta
IRAK4: Interleukin-1 receptor-associated kinase-4
LPS: Lipopolysaccharide
MyD: Myeloid differentiation factor
NEMO: Nuclear factor-kappa B essential modulator
NOD: Nucleotide-binding oligomerization domain
NLR: NOD-like receptor
NK: Natural killers
PAMP: Pathogen-associated molecular pattern
PGN: Peptidoglycan
PRR: Pattern recognition receptor
RIPK2: Receptor-interacting serine-threonine protein kinase 2
TLR: Toll-like receptor
TNF: Tumour necrosis factor
TAK1: TGF-beta-activated kinase
PID: Primary immunodeficiencies
